# Inhibition of MMP-2 and MMP-9 decreases cellular migration, and angiogenesis in in vitro models of retinoblastoma

**DOI:** 10.1186/s12885-017-3418-y

**Published:** 2017-06-20

**Authors:** Anderson H. Webb, Bradley T. Gao, Zachary K. Goldsmith, Andrew S. Irvine, Nabil Saleh, Ryan P. Lee, Justin B. Lendermon, Rajini Bheemreddy, Qiuhua Zhang, Rachel C. Brennan, Dianna Johnson, Jena J. Steinle, Matthew W. Wilson, Vanessa M. Morales-Tirado

**Affiliations:** 1 Department of Ophthalmology, Hamilton Eye Institute, the University of Tennessee Health Science Center, 930 Madison Ave, Room 756, Memphis, TN 38163 USA; 2 Department of Microbiology, Immunology and Biochemistry, the University of Tennessee Health Science Center, Memphis, TN USA; 3 Department of Oncology, St. Jude Children’s Research Hospital, Memphis, TN USA; 4 Department of Surgery, St. Jude Children’s Research Hospital, Memphis, TN USA; 5 Department of Anatomy and Cell Biology, Wayne State University, Detroit, MI USA

**Keywords:** Matrix metalloproteinases, MMP-2, MMP-9, Retinoblastoma, Therapy, Metastasis, VEGF, TGF-β1

## Abstract

**Background:**

Retinoblastoma (Rb) is the most common primary intraocular tumor in children. Local treatment of the intraocular disease is usually effective if diagnosed early; however advanced Rb can metastasize through routes that involve invasion of the choroid, sclera and optic nerve or more broadly via the ocular vasculature. Metastatic Rb patients have very high mortality rates. While current therapy for Rb is directed toward blocking tumor cell division and tumor growth, there are no specific treatments targeted to block Rb metastasis. Two such targets are matrix metalloproteinases-2 and -9 (MMP-2, −9), which degrade extracellular matrix as a prerequisite for cellular invasion and have been shown to be involved in other types of cancer metastasis. Cancer Clinical Trials with an anti-MMP-9 therapeutic antibody were recently initiated, prompting us to investigate the role of MMP-2, −9 in Rb metastasis.

**Methods:**

We compare MMP-2, −9 activity in two well-studied Rb cell lines: Y79, which exhibits high metastatic potential and Weri-1, which has low metastatic potential. The effects of inhibitors of MMP-2 (ARP100) and MMP-9 (AG-L-66085) on migration, angiogenesis, and production of immunomodulatory cytokines were determined in both cell lines using qPCR, and ELISA. Cellular migration and potential for invasion were evaluated by the classic wound-healing assay and a Boyden Chamber assay.

**Results:**

Our results showed that both inhibitors had differential effects on the two cell lines, significantly reducing migration in the metastatic Y79 cell line and greatly affecting the viability of Weri-1 cells. The MMP-9 inhibitor (MMP9I) AG-L-66085, diminished the Y79 angiogenic response. In Weri-1 cells, VEGF was significantly reduced and cell viability was decreased by both MMP-2 and MMP-9 inhibitors. Furthermore, inhibition of MMP-2 significantly reduced secretion of TGF-β1 in both Rb models.

**Conclusions:**

Collectively, our data indicates MMP-2 and MMP-9 drive metastatic pathways, including migration, viability and secretion of angiogenic factors in Rb cells. These two subtypes of matrix metalloproteinases represent new potential candidates for targeted anti-metastatic therapy for Rb.

**Electronic supplementary material:**

The online version of this article (doi:10.1186/s12885-017-3418-y) contains supplementary material, which is available to authorized users.

## Background

Retinoblastoma (Rb) is the most common primary intraocular tumor in children with an incidence of approximately 12 cases per million children under 4 years of age in the United States [1]. Mutation of the tumor suppressor gene, *RB1*, can lead to the disease sporadically or through inheritance. Germline mutations of *RB1* account for approximately 40% of cases and exhibit an autosomal dominant pattern of inheritance [2]. Germline *RB1* often affects both eyes whereas the more common sporadic form of the disease is often unilateral and accounts for 60% of all cases [2]. If diagnosed early, intraocular retinoblastoma can be effectively treated; however, the more advanced disease can metastasize to the central nervous system (CNS) in which case, mortality rates are greatly increased [3]. Initial tumor invasion from the retina to the sclera and post laminar optic nerve often pre-stages CNS metastasis and is indicative of high risk for later CNS metastasis [3]. Clinical risk factors that increase the incidence of metastasis in these patients include older age [4–6], laterality [7], vascularity [8, 9], and stage present upon diagnosis [10].

The dissemination of malignant neoplasms is assumed to require degradation of different components of the matrix and basement membrane. Matrix metalloproteinases (MMPs) are responsible for degradation of a number of extracellular matrix (ECM) components. There are over 20 recognized MMPs, each with specific substrate requirements and structural domains [11–13]. Among these are two highly associated with tumor dissemination and invasiveness [14, 15]: MMP-2 (*aka* gelatinase A) and MMP-9 (*aka* gelatinase B), which degrade type IV collagen and gelatin substrates. Cumulative work in different solid tumors has generated great interest in the development of MMP inhibitors (MMPI) as potential therapeutic anti-metastatic agents. Some synthetic MMPI have been tested in clinical trials in solid tumors other than Rb and show different levels of efficacy [16, 17]. Recent Clinical Trials by Gilead Sciences are evaluating MMP activity in different solid tumors, including non-small cell lung carcinoma (NSCLC), pancreatic adenocarcinoma, colorectal cancer (CRC) and breast cancer, and their effect in the tumor microenvironment by using an anti-MMP-9 therapeutic antibody [18]. The antibody, GS-5745 [19], is a humanized monoclonal antibody against MMP-9, which upon binding MMP-9 results in inhibition of ECM degradation and possibly a reduction in tumor growth and risk of metastasis. Immunohistochemical analysis of primary Rb tumors show that MMP-2 and MMP-9 protein levels are higher in samples that had invaded the optic nerve [20, 21]. To our knowledge, the effects of MMPI on Rb have not been analyzed comprehensively in vitro*.* Here, we provide a detailed analysis of two MMPI on cellular viability, levels of pro-angiogenic factors, migration and immunomodulatory proteins in two well-studied Rb cell lines: Y79 and Weri-1. These two Rb cell lines have somewhat different characteristics, with Y79 exhibiting inherent metastatic properties and Weri-1 exhibiting non-metastatic properties. Our aim was to examine responses of both cell lines since it is likely that Rb tumors in vivo may contain mixed populations of tumor cells with varying metastatic potential. Our results demonstrate that pharmacological inhibition of MMPs reduces Rb cell viability, migration, and secretion of the pro-angiogenic factors VEGF and Angiopoietin-2 in either one or both types of Rb cell lines. These promising findings provide an impetus for future in vivo studies to evaluate MMPI as a potential adjunct therapy for Rb patients.

## Methods

### Cell lines, growth media and tissue culture

Y79 (ATCC-HTB-18) [22], Weri-1 (ATCC-HTB-169) [23], Retinoblastoma (Rb) tumor cell lines were purchased from the American Type Culture Collection (ATCC, Manassas, VA). Cells were grown in RPMI-1640 (MediaTech, Herndon, VA) supplemented with 10% Fetal Bovine Serum (Hyclone, Logan, UT), 1% of Penicillin G Sodium Salt/Streptomycin Sulfate (100X) (Lonza). Rb cell lines were grown under different conditions, including ARP100 (MMP-2 inhibitor, Santa Cruz Biotechnology) at 5 μM and AG-L-66085 (MMP-9 inhibitor, Santa Cruz Biotechnology) at 5 μM concentration, unless otherwise specified. Incubation proceeded overnight at 37 °C/5%CO_2_. The IC_50_ values for ARP100: MMP-2: 12 nM; MMP-3: 4.5 μM; MMP-7: 50 μM. The IC_50_ values for AG-L-66085: MMP-9: 5 nM; MMP-1: 1.05 μM.

## qPCR analyses

### RNA isolation

RNA from 2.5 × 10^6^ Rb cells was extracted following the Qiagen® miRNeasy Mini Kit (Qiagen, Valencia, CA) manufacturer’s recommendations. Cells were lysed and homogenized prior to addition of chloroform. The upper colorless phase was transferred to a clean tube after centrifugation followed by 100% ethanol precipitation. The extract was passed through a spin column followed by on-column DNase digestion. The column membrane was washed with RNase free water for RNA elution. RNA concentration was assessed by analysis on Nanodrop Spectophotometer.

### cDNA synthesis and pre-amplification

Synthesis of cDNA was performed using the SuperScript® VILO™ cDNA Synthesis Kit (Life Technologies, Grand Island, NY). Following manufacturer’s directions we used 100 ng of RNA and combined them with Reaction Buffer and Enzyme Mix. Material was pre-amplified using TaqMan® PreAmp Master Mix as before [24] and the primers analyzed to use minimal amounts of material while increasing sensitivity of detection. The reaction was kept at −20 °C until ready to use.

### PCR

We used the following Human TaqMan® Gene Expression Assays: *HPRT1* (Hs02800695_m1), *MMP2* (Hs01548727_m1), *MMP7* (Hs01042796_m1), *MMP9* (Hs00234579_m1), *MMP14* (Hs01037003_g1) all from Life Technologies (Grand Island, NY). A final volume of 10 μL was loaded into each well after combination of TaqMan® Universal Master Mix, cDNA, primers and Nuclease Free water. Plates were run using Roche® LightCycler 480 and data were analyzed using the Comparative Ct Method as in [24, 25].

## siRNA experiments

Y79 Rb cells were plated overnight in 6-well plates at a cell density of 2.5 × 10^5^ cells per well in 2 mL RPMI/10% FBS (no antibiotics) final volume. Two solutions were made: solution A contained 0.75 μg of siRNA into 100 μL of siRNA Transfection Medium (Santa Cruz Biotechnology) per well; solution B contained 6 μL of siRNA Transfection Reagent into 100 μL siRNA Transfection Medium. Silencers: *MMP2*: sc-29,398; *MMP9*: sc-29,400; both from Santa Cruz Biotechnology. Solutions A and B were mixed and incubated at RT for 30 min. Cells were harvested and washed in siRNA Transfection Medium. We proceeded to resuspend harvested cells in 800 μL of siRNA Transfection Medium per well. Added the mixture of solutions A and B onto the cells, mixed gently and incubated for 24 h at 37 °C/5%CO_2_. Next, we added 1 mL of RPMI/20%FBS without removing the transfection mixture and incubated cells for an additional 24 h prior to performing functional assays. As a control, we used a scramble sequence that does not lead to degradation of any known cellular mRNA.

## Protein assessment

### Enzyme-linked immunosorbent assays (ELISA)

Human MMP-2, human MMP-9, human VEGF, and universal TGF-β1 ELISA kits were purchased from Life Technologies. Human Angiopoietin-2 was purchased from Sigma-Aldrich (St. Louis, MO). All assays used manufacturer’s instructions. Biological replicates of cell lysates (25 μg for MMP-2 and MMP-9; 40 μg for VEGF and TGF-β1) were assayed in triplicates. After the addition of the samples, all plates were incubated on a shaker at RT for 2-h, according to instructions. Plates were washed and incubated with their Biotin Conjugate on a shaker for 1-h at RT followed by addition of Streptavidin-HRP at RT for 30-min. In the TGF-β1 Kit, these two steps were combined for a 3-h incubation as indicated by the protocol. Afterwards, 100 μL of stabilized chromogen were added to each well and incubated in the dark for 30-min at RT followed by addition of stop solution prior to measuring O.D. at 405 nm.

### Western blot assays

Cells were lysed in RIPA Buffer (Life Technologies) as previously described [26]. Protein concentrations were calculated using the Pierce™ BCA Protein Assay Kit (Thermo Scientific). A total of 50 μg of denatured protein was used for each sample loaded in a Bolt™ 4–12% Bis-Tris Plus Gel (Invitrogen), following manufacturer’s instructions. Membrane was blocked in 20 mL of Pierce™ Fast Blocking Buffer followed by incubation with antibodies. Primary antibodies used: MMP-2 (D8N9Y) rabbit monoclonal antibody at 1:1000, MMP-9 rabbit polyclonal antibody at 1:1000, E2F rabbit polyclonal antibody at 1:1000, and β-Actin (D6A8) rabbit monoclonal antibody HRP conjugated at 1:1000. Secondary antibody was Anti-rabbit IgG, HRP-linked at 1:2000. All antibodies were from Cell Signaling Technologies® (Danvers, Massachusetts, USA). We used the Biotinylated Protein Ladder Detection Pack (Cell Signaling Technologies®), which includes the biotinylated protein ladder and the anti-biotin, HRP-linked antibody. SuperSignal West Pico Chemiluminiscent Substrate (Thermo Scientific) was used to develop the signal. Densitometry analysis was done using Kodak Molecular Imager, as previously done [27–29].

## Cellular proliferation

Quantitation of cell proliferation and viability was performed through use of CellTiter 96® AQ_ueous_ Non-Radioactive assay (MTS) (Promega, Madison, Wisconsin, USA) following manufacturer’s suggested guidelines. Briefly, 5.0 × 10^4^ Y79 and Weri-1 Rb cell lines were cultured per well under different culture conditions: untreated, MMP2I, and MMP9I. CellTiter 96® AQ_ueous_ was added at a concentration of 10 μL of reagent per 100 μL volume per well at specific time points of 0-, 48-, 72-, 96- and 120-h after culture. After addition of CellTiter reagent, cells were incubated at 37 °C/5% CO_2_ for an additional 2-h before absorbance was read at 485 nm using 630 nm as a reference.

## Cell cycle

Y79 cells were plated under different cell culture conditions overnight at 37 °C/5% CO_2_. Next day cells were then harvested and fixed in PBS/2% paraformaldehyde (PFA) for 15 min on ice, then washed and permeabilized using 0.1% Triton™ X-100 (Sigma-Aldrich) for 20 min. We used far-red fluorescent DNA dye, DRAQ5™ (BioLegend, San Diego, CA, USA), at a 1:100 concentration in PBS/1% FBS for 15 min on ice to assess cell cycle progression. This is a cell-permeant DNA binding anthraquinone dye, which intercalates between adenine and thymine (A-T) bases of double stranded DNA. DRAQ5™ was excited at 642 nm and acquired using a 642 to 740 nm filter on the Amnis FlowSight® imaging cytometer (Amnis Corporation, EMD Millipore, Seattle, WA, USA). Data was acquired and analyzed by INSPIRE and IDEAS v6.2 softwares, respectively (Amnis Corporation).

## Migration and invasion assays

### Migration/ wound healing assay

CytoSelect™ 24-well Would Healing Assay kit was purchased from Cell Biolabs Inc. (San Diego, CA). The 24-well plate was pretreated with 500 μL of 0.1 mg/mL Poly-L-Lysine hydrobromide (Sigma-Aldrich) per manufacturer’s instructions and incubated at 37 °C for 1-h. Wells were washed with distilled sterile water twice and dried in the biosafety cabinet for 2-h. We added 500 μL of 1X attachment factors (Life Technologies) containing gelatin (substrate of both MMP-2 and MMP-9) per well and incubated at 37 °C for 30 min. Solution was aspirated and replaced by Rb cells at a concentration of 1.0 × 10^6^ cells/mL. Cell culture conditions included untreated, MMP2I, and MMP9I. We ensured cells were evenly distributed and incubated the plate at 37 °C to create a 95% confluent monolayer of cells. The inserts were removed; wells were washed twice with distilled sterile water to remove unattached cells and debris. The cells were then resuspended in 500 μL of respective culture conditions. Pictures were taken and 0-, 2-, 6-, 24-, and 48-h time points and analyzed for cell migration using an Axiovert 40 CFL (Zeiss, Germany) at a 12.5× total magnification (lens 2.5×, objective 10×, and camera 0.5×).

### Invasion assay

CytoSelect™ Cell Invasion Assay kit was purchased from Cell Biolabs Inc. We use an 8 μm pore polycarbonate membrane coated with basement membrane matrix solution. Rb cell suspension (serum free media) was placed in the upper chamber to determine the invasion capacity of the cells after degradation of the matrix membrane proteins 6 h post culture. Invasive cells were stained and quantified with a light microscope under 100× total magnification (lens 2.5×, objective 40×), with 4 individual fields per insert. Inserts were placed to wells containing 200 μL of Extraction Solution followed by 10 min incubation at RT on an orbital shaker. Quantitation of cells measured at OD 560 nm using spectrophotometer.

### Statistical analysis

Data on bar graphs are expressed as means ± SD or ± SEM (as indicated), with *p* < 0.05 considered statistically significant. The data were compared where appropriate by paired Student t test or by the Holm-Sidak Method, with alpha = 5.0%.

## Results

### Inhibition of MMP-2 and MMP-9 decreases migration in the metastatic Y79 Rb cell line, and viability in the non-metastatic Weri-1 model

**Fig. 1:**
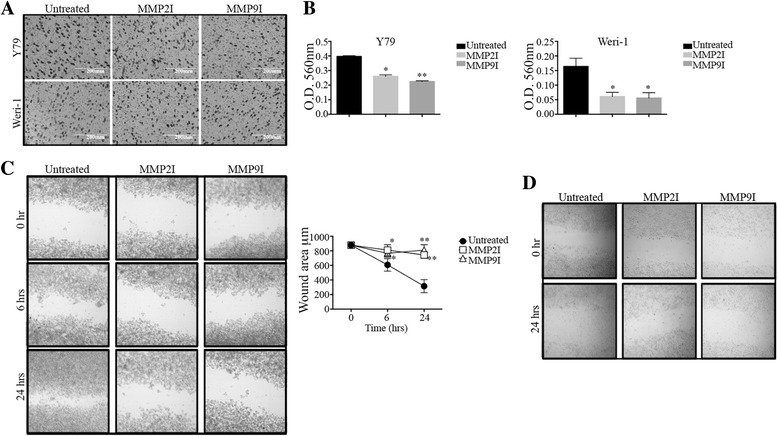
Inhibition of MMP-2 and MMP-9 reduced Rb migration. **a**-**b** Y79 and Weri-1 cells were added to the upper chamber of an 8 μm polycarbonate membrane coated with basement membrane proteins in serum free media. The lower chamber contained cell culture media with or without MMPI. Six-hours post culture, invasive cells degraded the ECM and were collected, stained and counted. Representative figures are shown in **a** with a 100× total magnification. Cells were extracted and OD measured in **b** *left* for Y79 and *right* for Weri-1. **c** Y79 Rb cells were cultured in the presence or absence of MMP-2 or MMP-9 inhibitors for 48-h on poly-L-lysine coated wells with gelatin as substrate. Sterile in-well inserts created a gap of 900 μm. Gap closure was recorded at different time intervals using an Axiovert 40 CFL. Total magnification is 12.5×. Plotted results are in **c** *right*. **d** Weri-1 cells showed increased cell death and detachment from coated surface. For each condition *n* = 3; gap was measured in 5 different points

Tumor migration and invasion of the optic nerve and the uvea has a significant impact in the prognosis of Rb. To investigate the effects of inhibition of MMP-2 and MMP-9 on the migration of Rb cells we used both a metastatic model represented by the Y79 cell line and a non-metastatic model, represented by the Weri-1 cell line. Cells were added to the upper chamber of an 8 μm polycarbonate membrane coated with basement membrane proteins in serum free media. The lower chamber had media in the presence or absence of the MMPI. We used ARP100 as an inhibitor of MMP-2 at a 5 μM concentration; and AG-L-66085 as a MMP-9 inhibitor at a 5 μM concentration, as previously described [30]. Our results showed a significant reduction of Rb cell migration through the basement membrane, or extracellular matrix (ECM), suggesting MMP-2 and MMP-9 activity are necessary to degrade ECM and promote cellular invasion in Rb. In Fig. 1a we show a representative field for each insert. Quantitation analyses shown in Fig. 1b show statistical difference between untreated Y79 and those treated with the MMPI (Y79 Rb cells, Untreated versus MMP2I: 0.397 ± 0.06 versus 0.260 ± 0.010, *p* = 0.01; versus MMP9I: 0.225 ± 0.005, *p* = 0.0009; Weri-1 Rb cells, Untreated versus MMP2I: 0.164 ± 0.028 versus 0.061 ± 0.014, *p* = 0.043; versus MMP9I: 0.056 ± 0.018, *p* = 0.0294). Next, we adhered Rb cells to poly-L-lysine hydrobromide coated surfaces and created artificial wounds of approximately 900 μm. The closure of the gap area was measured at different time intervals for up to 48-h. We observed Y79 untreated cells closed the gap area (Fig. 1c), while MMP2I and MMP9I-treated Y79 cells showed a significant reduction in migration (Untreated versus MMP2I at 24 h: 315 ± 45 versus 742.5 ± 22.5, *p* = 0.0001; versus MMP9I: 810 ± 36.7, *p* = 0.0001). Migration potential as measured by the wound-healing assay revealed that inhibition of either MMP-2 or MMP-9 caused a significant reduction of Y79 cells migration. Cellular viability assays (Additional file 1: Figure S1) showed both MMP2I and MMP9I significantly reduced the viability of Y79 cells (Untreated versus MMP2I: 116.67% ± 1.40 versus 42.66% ± 1.4, *p* < 0.005; versus MMP9I: 32% ± 0, *p* < 0.005). In addition to the cytotoxic effect we observed a significant increase in the percentage of cells within the G0/G1 cell cycle phase in Y79 cells treated with MMP9I compared to those untreated (Additional file 1: Figure S1, Untreated versus MMP9I: G0/G1 phase: 32.44% ± 0.907 versus 49.51 ± 1.059; S phase: 5.23% ± 0.165 versus 5.28% ± 0.062; G2/M phase: 5.16% ± 0.117 versus 4.252% ± 0.335).

We were unable to carry out the migration assay using Weri-1 cells because these cells detached from the surface of the wells after treatment with either of the inhibitors (Fig. 1d), which precluded any meaningful measurement. To better understand this we did a titration assay (500 nM to 25 μM range) of the MMPI to investigate the sensitivity of Weri-1 Rb cells to MMP2I (*left*) and MMP9I (*right*). Results shown in Additional file 2: Figure S2 revealed Weri-1 Rb cells are sensitive to inhibitors even at low concentrations.

Collectively, these findings support the conclusion that MMP-2 and MMP-9 activity stimulates Rb cell migration in vitro and that similar pathways could be involved in Rb metastasis in vivo.

### Downregulation of MMP-2 and MMP-9 by pharmacological inhibitors in Y79 cells

**Fig. 2:**
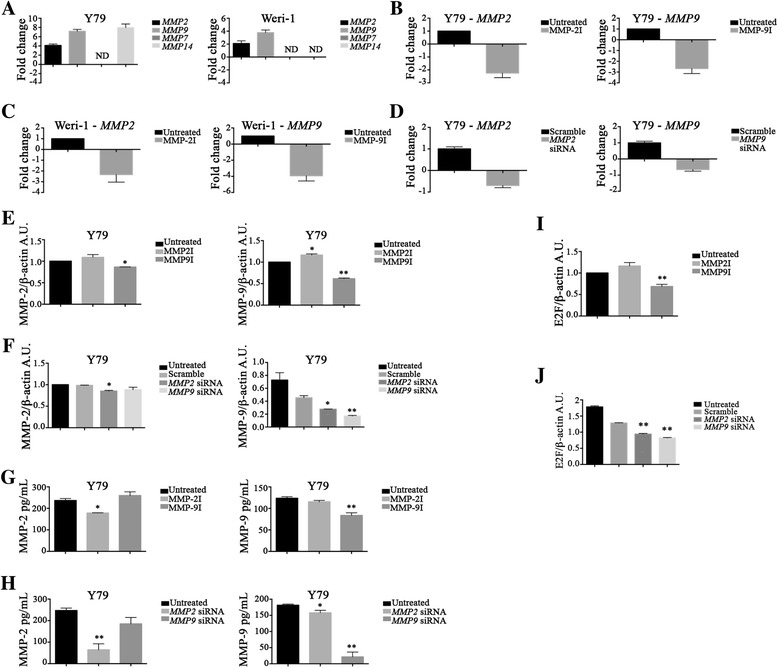
Pharmacological inhibitors of MMP-2 and MMP-9 downregulate *MMP2* and *MMP9* mRNA. **a** The following MMPs were examined at the transcriptional level: *MMP2*, *MMP7*, *MMP9*, and *MMP14*. Y79 (*left*) and Weri-1 (*right*) cells were harvested for RNA isolation and cDNA synthesis. Material was pre-amplified using the TaqMan® PreAmp Master Mix with the respective primers. qPCR was done and results show mRNA expression relative to *HPRT1* as endogenous control. Bar graphs indicate results ±SD; *n* = 3 biological replicates in triplicates. Y79 Rb cells express *MMP2*, *MMP9* and *MMP14*; Weri-1 expressed *MMP2* and *MMP9*. **b**-**c** Y79 (**b**) and Weri-1 (**c**) cells were treated with MMP-2 and MMP-9 inhibitors overnight. RNA and cDNA was extracted as in **a** showing that the inhibitors act at the transcriptional level. Bar graphs indicate fold change ±SD; *n* = 3. Standard deviation obtained from the biological replicates. **d** Knockdown of *MMP2* and *MMP9* by RNA interference shows on-target effects. Downregulation of *MMP2* and *MMP9* after siRNA compared to scramble samples. qPCR done as in **a**. **e**-**f** Reduction of MMP-2 and MMP-9 protein in Rb cells treated with MMPI (**e**) and siRNA (**f**); *p < 0.05, **p < 0.005. Western blot bar graphs indicate results ±SEM ratio of target protein to β-actin; *n* = 3. **g**-**h** ELISA analyses of MMP-2 and MMP-9 protein of whole cell lysates after treatment with MMPI (**g**) or siRNA (**h**); *p < 0.05, **p < 0.005. **i**-**j**, E2F regulates MMP expression in Y79 cells. Y79 cells treated with MMPI (**i**) or with siRNA (**j**) were assessed by Wb analysis for E2F. Western blot bar graphs indicate results ±SEM ratio of target protein to β-actin; *n* = 3; **p < 0.005

In Fig. 1a we investigated MMP-2 and MMP-9 activity in migration behavior. We hypothesized that Y79, considered the metastatic model for Rb [31], has higher levels of *MMP2* and *MMP9* at mRNA and protein levels compared to the non-metastatic Weri-1. Qualitative PCR analysis shown in Fig. 2a revealed Y79 had higher expression of both *MMP2* and *MMP9* mRNA transcripts compared to Weri-1, as we hypothesized (Y79, *MMP2*: 4.116 ± 0.3, *MMP9*: 7.186 ± 0.4; Weri-1, *MMP2*: 2.1 ± 0.4, *MMP9*: 3.78 ± 0.4). Additional analyses were performed to investigate if other MMPs associated with tumor invasion were expressed in these Rb cell lines. We found no detection (ND) of *MMP7* mRNA, but found expression of *MMP14* (7.96 ± 0.8) in Y79 cells. Given the recent emphasis in the role of MMP-2 and MMP-9 in ECM degradation and cancer invasion we are focusing our studies on investigating MMP-2 and MMP-9 activity in Rb.

MMP regulation occurs primarily at the transcriptional level. Next, we verified the effectiveness of the used MMPI in downregulation of MMP gene expression in both Rb models. As shown in Fig. 2b, there was a significant reduction in the mRNA expression of both *MMP2* and *MMP9* by their respective inhibitors in Y79 cells. Similar results were found in Weri-1 cells (Fig. 2c). These results confirmed that MMPI inhibited MMP function by downregulation of *MMP2* and *MMP9* mRNA expression. Due to our laboratory’s interests in invasion and tumor aggressiveness we concentrated the rest of our investigations on Y79, the more aggressive and metastatic Rb tumor model. Despite inhibition of *MMP2* mRNA, we still observed intracellular protein by Western blot (Wb) analysis (Fig. 2e), but a significant reduction by ELISA (Fig. 2g, Untreated versus MMP2I: 237 ± 9 versus 179 ± 10, *p* < 0.005; versus MMP9I: 260 ± 17, *p* = 0.266). The difference could stem from the specificity of the assays, as the ELISA measures active enzyme and the Wb measured total protein. However, treatment with MMP9I showed a significant reduction in MMP-9 intracellular protein by both Wb and ELISA (Fig. 2e and g, Untreated versus MMP2I: 124 ± 3 versus 115 ± 3, *p* = 0.106; versus MMP9I: 84 ± 6, *p* < 0.0005).

E2F belongs to a family of transcription factors that regulate cell cycle and DNA replication in mammalian cells [32]. We investigated the expression of E2F in Y79 Rb cells and if treatment with MMPI could modulate their levels. As shown in Fig. 2i, there is a significant reduction of E2F levels in Y79 cells treated with MMP9I, but not MMP2I, suggesting E2F regulates MMP-9 expression. Next, we investigated if this was an on-target effect of the MMP9I by using siRNA. We targeted *MMP2* and *MMP9* and confirmed downregulation of their gene expression and proteins levels (Fig. 2d–h). The results in Fig. 2j showed a significant reduction in E2F levels by both *MMP2* and *MMP9* siRNA compared to the scramble group, suggesting this is not an off-target effect of downregulation of the MMP-2 and MMP-9.

### Pharmacological inhibition of MMPs reduces secretion of angiopoietin-2, but not VEGF, in Y79 cells

**Fig. 3:**
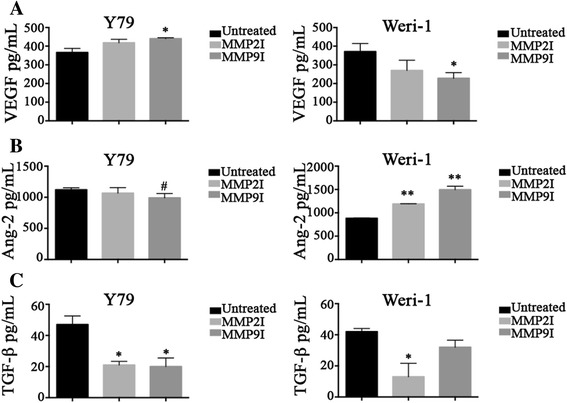
MMP inhibition reduces angiogenic protein levels. Y79 and Weri-1 cells were cultured in the presence or absence of the MMPI overnight. Next day, we collected cell lysates (**a**-**b**) and supernatants to investigate protein levels by ELISA. **a** shows VEGF protein levels; **b** shows Ang-2 protein levels and **c**, shows levels of TGF-β1, an immunomodulator. In all secretion analyses bar graphs indicate results ±SD; *n* = 3; *p < 0.05, **p < 0.005, #*p* = 0.05

Retinoblastoma tumors are highly angiogenic. Aqueous humor from enucleated Rb eyes has been shown to trigger significant angiogenic activity [33]. One key angiogenic factor is vascular endothelial growth factor (VEGF), shown by Hollborn and colleagues [34] to stimulate MMP-9 production in human retinal pigment epithelial cells. To further examine possible mechanisms by which MMPs might stimulate migration and other pro-metastatic processes in Rb disease, we analyzed the effects of MMP inhibition on production of angiogenic factors, including VEGF and Angiopoietin-2. As shown in Fig. 3a *left*, there was no significant reduction in VEGF secretion in Y79 cells after treatment with MMP2I, but there was a significant increase when MMP9I was used (Untreated versus MMP2I: 366 ± 44 pg/mL versus 418 ± 37 pg/mL; *p* = 0.83; versus MMP9I: 440 ± 10 pg/mL; *p* = 0.01;). Holash and colleagues [35] reported that both VEGF and Angiopoietin-2, or perhaps the equilibrium between the two, influence tumor growth and vascular regression, prompting us to measure the effects of MMPI on Angiopoietin-2. The protein levels of Angiopoietin-2 in Y79 were reduced, although marginally significant, by MMP9I (Fig. 3b *left*: Y79 Untreated versus MMP2I: 1120.3 ± 65 pg/mL versus 1067.6 ± 153 pg/mL, *p* = 0.552; versus MMP9I: 990 ± 90 pg/mL, *p* = 0.05). In contrast, as shown in Fig. 3a *right*, the non-metastatic Rb cell line Weri-1 showed a significant reduction in VEGF after MMP9I treatment (Untreated versus MMP2I: 371 ± 75 pg/mL versus 270 ± 95 pg/mL, *p* = 0.221; versus MMP9I: 228 ± 60 pg/mL; *p* = 0.005) but a significant increase in Angiopoietin-2 (Untreated versus MMP2I: 883 ± 10 versus 1190 ± 13, *p* < 0.005; versus MMP9I: 1495 ± 147, *p* < 0.005) after treatment (Fig. 3b *right*). Collectively, these results showed that in the metastatic Y79 cell line, we observed a significant increase in VEGF by MMP9I, and a reduction, albeit minimal in Angiopoietin-2 (*p* = 0.05). The opposite was observed in Weri-1, as there was a significant reduction in VEGF by MMP9I and a significant increase in Angiopoietin-2 by MMP2I and MMP9I. These results highlight the complexity associated with Rb disease.

Transforming Growth Factor-beta 1 (TGF-β1) is a potent immunosuppressor of cytotoxic cells by depressing cytolytic ability and thus promoting metastases. Recent work suggests MMPs may stimulate TGF-β1 activity [26, 32, 36]. To determine if inhibition of MMP-2 or MMP-9 could affect the TGF-β1 pathway in Rb, we measured secretion of TGF-β1 by Y79 cells after treatment with the inhibitors. As shown in Fig. 3c *left*, TGF-β1 secretion was significantly reduced in Y79 cells by either of the inhibitors (Untreated versus MMP2I: 47.0 ± 11 pg/mL versus 20.0 ± 4 pg/mL, *p* = 0.010; versus MMP9I: 20.7 ± 11 pg/mL, *p* = 0.013). Similarly, we tested TGF-β1 secretion in Weri-1 cells (Fig. 3c *right*) and found it was significantly reduced after MMP-2 inhibition (Untreated versus MMP2I: 42.0 ± 4 pg/mL versus 13.2 ± 15 pg/mL, *p* = 0.012), but not MMP-9 inhibition (Untreated versus MMP9I: 32 ± 9 pg/mL, *p* = 0.088). Here, we demonstrated the convolution associated with metastatic and non-metastatic Rb cell lines. We found MMP-2 and MMP-9 exert direct activity on the angiogenesis, production of TGF-β1 and migration in Rb cell lines.

## Discussion

Our work focuses on MMP-2 and MMP-9 activity in Rb, the most common intraocular malignancy in children. Consistent with previous reports, we show MMP-2 and MMP-9 are present in Rb cell lines. For the first time in retinoblastoma, we provide a comprehensive in vitro analysis of two cell lines, Y79 and Weri-1, which represent the metastatic and non-metastatic model for Rb. As part of our in depth analysis we compared both cell lines in their response to several properties: invasion, cellular migration, mRNA expression and protein levels of MMP-2 and MMP-9, the production of the angiogenic factors VEGF and Angiopoietin-2, and the immunomodulatory protein TGF-β1.

The outcomes of our experiments revealed differences in several intrinsic properties associated with tumor progression in Y79 and Weri-1. Tumor cells in patients are likely to have diverse cell populations that have varying metastatic potential, thus studying both cell lines provides important insight into actual properties of tumor in vivo. While these two cell types both respond to MMPI, they do so in different ways using different pathways. The MMPI used in this study mediate their effect on Rb cells through inhibition of *MMP2* and *MMP9* mRNA in both Y79 and Weri-1. However, the effects on angiogenic factors differ between cell types. Our results suggest the mechanisms underlying the production of angiogenic factors are different among these cells. The production of VEGF in Weri-1 may be more dependent on MMP-2 or MMP-9 activity as there was a significant reduction in protein production after treatment with MMP2I and MMP9I. Conversely, production of Angiopoietin-2 is increased in Weri-1 after MMPI treatment suggesting Angiopoietin-2 production is independent of MMP-2 or MMP-9 activity. These results suggest these two angiogenic pathways are not involved in primary actions on metastasis, as Weri-1 is the non-metastatic model. In contrast, Y79 cells showed a significant increase in VEGF production after MMPI treatment, although MMP9I reduced Angiopoietin-2. This is of interest as Holash and colleagues [35] previously described the dynamic balance in vessel regression and tumor growth using a rat glioma model. Two key players in this model are angiopoietins (Ang) and VEGF. Co-expression and increase in both VEGF and Angiopoietin-2 are associated with blood vessel proliferation. According to the authors, if there is overexpression of one of these players, there is vessel destabilization and regression. Work from Zhu and colleagues [37] demonstrated that concomitant expression of VEGF and Angiopoietin-2 resulted in increased microvessel density in solid tumors [38] and cerebral angiogenesis. The co-expression of these angiogenic factors contributes to the induction of microvessel sprouting in vascular networks [39]. Collectively, our results show destabilization of angiogenic components, VEGF for Weri-1 and Angiopoietin-2 for Y79 Rb cells.

Transforming Growth Factor- beta 1 (TGF-β1) is a pleiotropic cytokine suggested to be the main inducer of tumor epithelial-to-mesenchymal (EMT) transition (reviewed in [40]) and to facilitate invasion by suppressing the host immune system [41, 42]. In this study we found TGF-β1 to be significantly reduced after MMP2I treatment in both Y79 and Weri-1 cells. Work from Kim and colleagues highlighted the role of this cytokine in upregulation of MMP-2 and MMP-9 in the MCF10A breast cancer cell line [43]; it is also known that these MMPs participate in TGFβ cleavage for further cytokine release. TGFβis the focus of other studies in the lab as it was demonstrated to be localized in proximity to tumor vasculature and to promote drug resistance [44].

## Conclusions

**Fig. 4:**
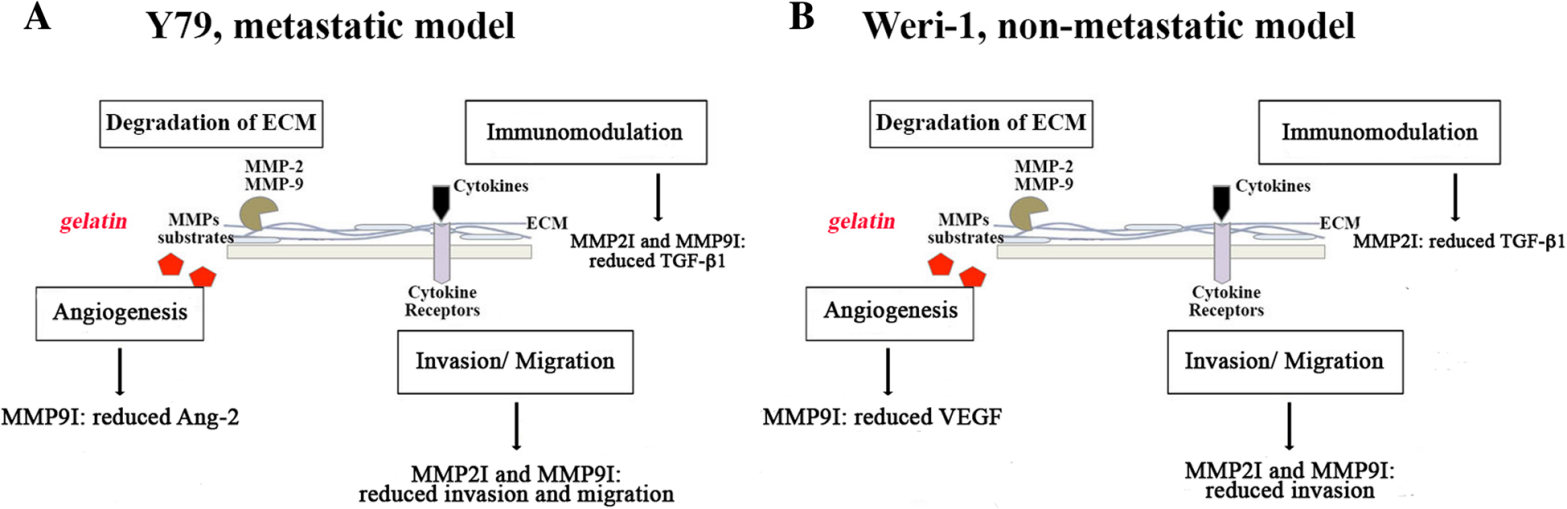
Working model of the roles of MMP-2 and MMP-9 in retinoblastoma cells. Y79 and Weri-1 cells represent the metastatic and the non-metastatic model for Rb, respectively. Our work shows differences in viability, migration and angiogenic-associated responses in Rb cells after inhibition of MMP-2 and MMP-9. **a** Y79 cells showed a profound defect in migration and invasion along with and a significant reduction in Angiopoietin-2 and TGF-β1 proteins. These results highlight Y79’s migratory and invasive potential, which may be dependent upon MMPs. **b** Analyses of Weri-1 cells show MMP-2 and MMP-9 are involved in multiple processes, including viability of cells and VEGF, as well as TGF-β1 production

Our work reveals differences in several intrinsic properties associated with tumor progression in two cell lines representing the metastatic and non-metastatic form of Rb, Y79 and Weri-1. Based on our findings we developed a working model shown in Fig. 4. In addition to the intrinsic differences in Y79 and Weri-1, MMP-2 and MMP-9 play different roles in these cells. MMP-2 and MMP-9 activity stimulate Rb cell migration in Y79 and contribute to cell viability in Weri-1 cells. Furthermore, MMP-9 activity plays a role in Angiopoietin-2 production in Y79. In contrast, MMP-2 and MMP-9 play additional roles in Weri-1 cells. More work is needed to follow up on these promising results. Taken together, we provide a comprehensive in vitro analysis of MMP-2 and MMP-9 activity in Rb in several checkpoints that are deregulated in cancer. Our findings provide initial mechanistic insights into the benefits of potential MMP adjunct therapy in Rb patients.

## Additional files

Additional file 1: Figure S1. Inhibition of MMP-2 or MMP-9 reduced Rb viability and cell cycle progression. a, Y79 cells were cultured in the presence or absence of the MMPI overnight. Next day, we collected cells and assessed viability by Trypan Blue exclusion. Chemical inhibition of Y79 with MMPI significantly reduced cell yield when compared to untreated cells. b, RNA interference was used to confirm on-target effects of MMPIs. Y79 were cultured in the presence of either *MMP2* or *MMP9* siRNA. *MMP2* and *MMP9* knockdown groups showed significant reduction in cell yield, illustrating an on-target effect of MMPI. c, Imaging flow cytometry analysis showed inhibition of MMP9 prevents progression of Rb cell division using nuclear DRAQ5™ labeling. Bar graphs indicate results ± SEM to control. **p < 0.005. (TIF 434 kb)

Additional file 2: Figure S2. Weri-1 Rb cells are sensitive to MMPI. Weri-1 cells were cultured in the presence or absence of MMPI. The MMPI were used at a concentration range of 500 nM to 25 μM for up to 120 h. MTS proliferation solution was added to each well at a concentration of 10 μL solution per 100 μL at specific time points (0-, 48-, 72-, 96-, and 120-h) and incubated at 37 °C/5%CO_2_ for 2 h prior to reading on an absorbance reader. Values represent are optical density (O.D.) ± SEM at 482 nm with a reference wavelength of 630 nm. (TIFF 374 kb)

## Abbreviations

*aka*: also known as
Ang-2: Angiopoietin-2
cDNA: complementary DNA
CNS: Central Nervous System
DNA: Deoxyribonucleic acid
ECM: Extracellular matrix
ELISA: Enzyme-Linked Immunosorbent Assay
*MMP2*: Matrix metalloproteinase-2 gene
MMP-2: Matrix metalloproteinase-2 protein
*MMP9*: Matrix metalloproteinase-9 gene
MMP-9: Matrix metalloproteinase-9 protein
MMPI: Matrix metalloproteinase inhibitor
mRNA: messenger Ribonucleic Acid
OD: Optical density
PCR: Polymerase Chain Reaction
qPCR: qualitative Polymerase Chain Reaction
Rb: Retinoblastoma
*RB1*: Retinoblastoma 1 gene
RNA: Ribonucleic acid
SD: Standard deviation
SEM: Standard error measurement
TGF-β1: Transforming Growth Factor- beta 1
VEGF: Vascular Endothelial Growth Factor
