# Supplementary figures and images for: Inhibition of MMP-2 and MMP-9 decreases cellular migration, and angiogenesis in in vitro models of retinoblastoma
Source: BMC Cancer. 2017 Jun 20;17:434. doi: 10.1186/s12885-017-3418-y (PMC5477686; doi:10.1186/s12885-017-3418-y)

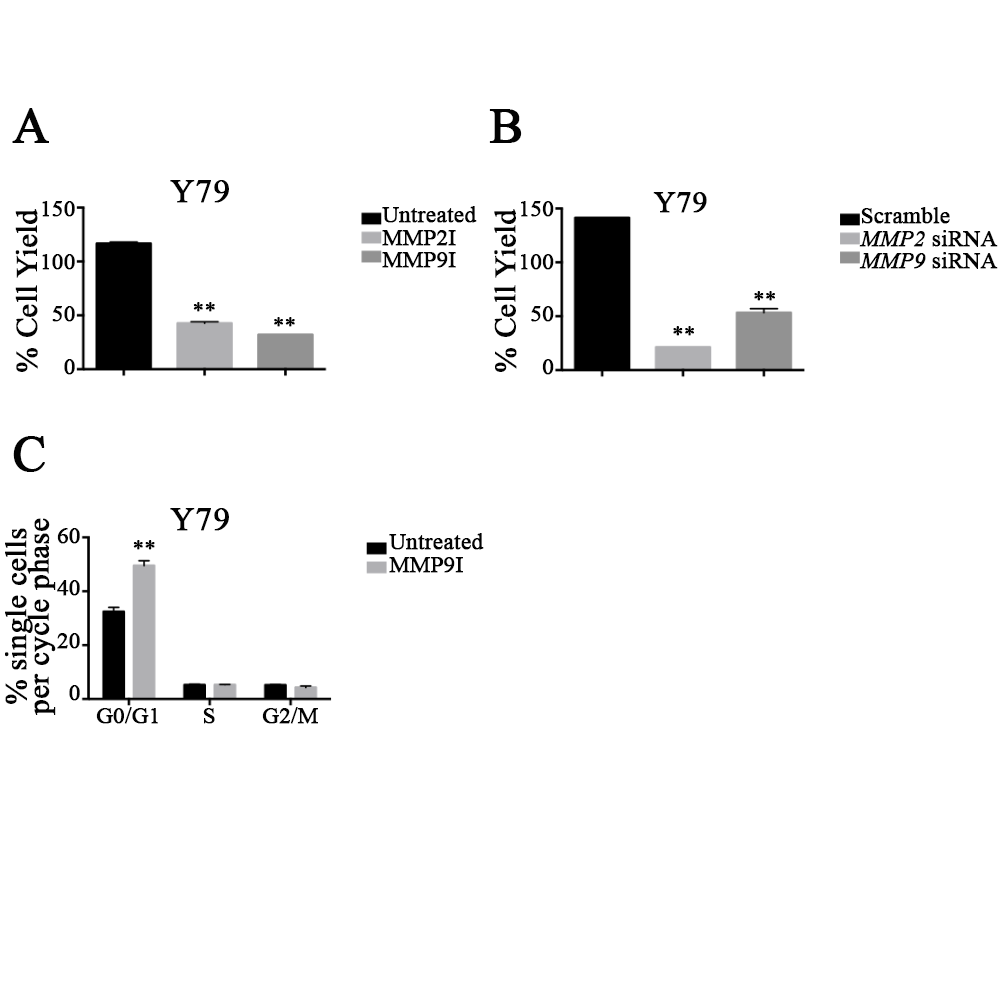

Supplement: Supplementary file 1 — Inhibition of MMP-2 or MMP-9 reduced Rb viability and cell cycle progression. a, Y79 cells were cultured in the presence or absence of the MMPI overnight. Next day, we collected cells and assessed viability by Trypan Blue exclusion. Chemical inhibition of Y79 with MMPI significantly reduced cell yield when compared to untreated cells. b, RNA interference was used to confirm on-target effects of MMPIs. Y79 were cultured in the presence of either MMP2 or MMP9 siRNA. MMP2 and MMP9 knockdown groups showed significant reduction in cell yield, illustrating an on-target effect of MMPI. c, Imaging flow cytometry analysis showed inhibition of MMP9 prevents progression of Rb cell division using nuclear DRAQ5™ labeling. Bar graphs indicate results ± SEM to control. **p < 0.005. (TIF 434 kb) [file 12885_2017_3418_MOESM1_ESM.tif]

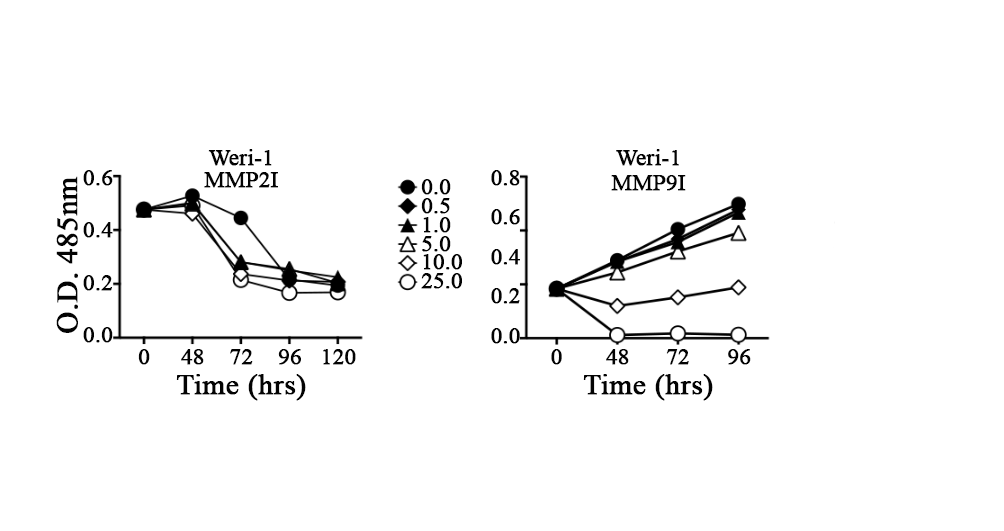

Supplement: Supplementary file 2 — Weri-1 Rb cells are sensitive to MMPI. Weri-1 cells were cultured in the presence or absence of MMPI. The MMPI were used at a concentration range of 500 nM to 25 μM for up to 120 h. MTS proliferation solution was added to each well at a concentration of 10 μL solution per 100 μL at specific time points (0-, 48-, 72-, 96-, and 120-h) and incubated at 37 °C/5%CO2 for 2 h prior to reading on an absorbance reader. Values represent are optical density (O.D.) ± SEM at 482 nm with a reference wavelength of 630 nm. (TIFF 374 kb) [file 12885_2017_3418_MOESM2_ESM.tif]
